# Simple Device for Fusing Low Heat Porcelain

**Published:** 1907-11

**Authors:** J. Allen Johnson

**Affiliations:** Middletown, Del.


### SIMPLE DEVICE FOR FUSING LOW HEAT PORCELAIN.

By Dr. J. Allen Johnson, Middletown, Del.

As many of our profession are located where the electric current is not available, the use of gas or gasoline for the fusing of porcelain bodies becomes a necessity. It is for the special benefit of that portion of the dental profession that I will present a method whereby the use of low fusing porcelain is simplified. In my own practice I only use the electric furnace for high fusing bodies.

Using 1-1000 matrix platinum, conform it to approximate the form of a straw hat having a conical crown. The crown diameter equal to a No. 18 disk, with a depth of ⅜ inch. A mica disk of size sufficient to cover the crown opening completes the oven part of the device. A support is made of thin iron wire and attached to work bench.

The tray should be readily removable from the support. This size of tray is large enough for any filling, and no investment need be used excepting for large contour fillings.

The mica disk excludes all gases from the blowpipe and, so far as I have observed, the results are equally as satisfactory as could be obtained with more elaborate outfits. The cost of construction is trifling, and good work may be done in less time than where the electric furnace is used.—Items of Interest.

